# Proteomic Analysis Reveals the Neurotoxic Effects of Chronic Methamphetamine Self-Administration-Induced Cognitive Impairments and the Role of Melatonin-Enhanced Restorative Process during Methamphetamine Withdrawal

**DOI:** 10.1021/acs.jproteome.3c00502

**Published:** 2023-09-07

**Authors:** Tanthai Polvat, Tanya Prasertporn, Piyada Na Nakorn, Supitcha Pannengpetch, Wilasinee Suwanjang, Jiraporn Panmanee, Sukhonthar Ngampramuan, Jennifer L. Cornish, Banthit Chetsawang

**Affiliations:** † Research Center for Neuroscience, Institute of Molecular Biosciences, Mahidol University, Salaya, Nakhon Pathom 73170, Thailand; ‡ Center for Research Innovation and Bioinformatics, Faculty of Medical Technology, Mahidol University, Salaya, Nakhon Pathom 73170, Thailand; § Center of Emotional Health, Department of Psychology, Macquarie University, Balaclava Road, North Ryde, NSW 2109, Australia

**Keywords:** methamphetamine, neurotoxicity, cognitive function, mitochondria, mitophagy, neuroplasticity, neurodegeneration, melatonin, drug addiction, proteomics

## Abstract

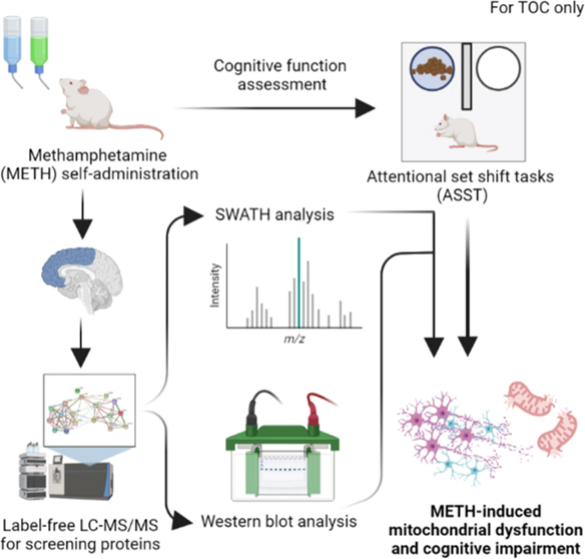

Cognitive flexibility is a crucial ability in humans that can be affected by chronic methamphetamine (METH) addiction. The present study aimed to elucidate the mechanisms underlying cognitive impairment in mice chronically administered METH via an oral self-administration method. Further, the effect of melatonin treatment on recovery of METH-induced cognitive impairment was also investigated. Cognitive performance of the mice was assessed using an attentional set shift task (ASST), and possible underlying neurotoxic mechanisms were investigated by proteomic and western blot analysis of the prefrontal cortex (PFC). The results showed that mice-administered METH for 21 consecutive days exhibited poor cognitive performance compared to controls. Cognitive deficit in mice partly recovered after METH withdrawal. In addition, mice treated with melatonin during METH withdrawal showed a higher cognitive recovery than vehicle-treated METH withdrawal mice. Proteomic and western blot analysis revealed that METH self-administration increased neurotoxic markers, including disruption to the regulation of mitochondrial function, mitophagy, and decreased synaptic plasticity. Treatment with melatonin during withdrawal restored METH-induced mitochondria and synaptic impairments. These findings suggest that METH-induced neurotoxicity partly depends on mitochondrial dysfunction leading to autophagy-dependent cell death and that the recovery of neurological impairments may be enhanced by melatonin treatment during the withdrawal period.

## Introduction

Cognitive flexibility is the ability to appropriately adapt and provide a behavioral response to new or unplanned information. It is comprised of several functions, such as working memory, to hold and manipulate necessary information while coordinating with attentional and self-inhibition processes. These brain processes support executive functions, such as decision making and goal planning, which can be modified in response to new environmental stimuli.^1^ Cognitive flexibility is a higher brain function regulated by multiple brain regions, particularly the prefrontal cortex (PFC). Therefore, impaired neuronal function in the PFC might cause impairment in this cognitive domain.^2^ Dopamine (DA) is an important neurotransmitter that regulates PFC function, and substantial evidence has indicated that the psychostimulant methamphetamine (METH) can disrupt the dopaminergic pathway by increasing DA overflow and DA auto-oxidation, resulting in reactive oxygen species (ROS) formation and mitochondrial dysfunction.^3,4^ Therefore, METH addiction may be a possible cause of cognitive dysfunction and neurodegeneration. Furthermore, recent studies have found that chronic METH exposure induced neurological dysfunction and behavioral and molecular changes within the brain.^5−7^ However, METH users who have been abstinent for at least 9 months showed substantial recovery from damage to the DA transporters but not from impairments in motor skills and memory.^8^ This evidence suggests that damage to DA transporter function may not underlie enduring neurological dysfunction, following METH use and points to the involvement of alternative mechanisms that could be targeted to enhance the recovery of neurological function during METH abstinence.

Recently, several lines of evidence demonstrated that mitochondrial dysfunction is crucial for inducing cell degeneration.^9^ It has been shown that mitochondrial stress initiates an excessive mitophagy process, resulting in autophagy-dependent neuron death.^10,11^ However, the effect of chronic METH treatment on these processes has yet to be elucidated. Intriguingly, melatonin, a neuroprotective compound with substantial evidence for its therapeutic efficacy to improve neuronal function, may mitigate the neurotoxic effects of METH treatment.^4,12,13^ Therefore, this study aimed to investigate the mechanisms underlying neuropathological processes and cognitive impairment in mice treated chronically with METH. In addition, the effect of melatonin treatment on recovery of neurological dysfunction and neurobehavioral deficits during METH withdrawal was also investigated. Cognitive function was evaluated in mice after chronic METH administration and METH withdrawal, and the effect of melatonin treatment during the withdrawal phase on cognitive function was also assessed. In addition, the profile of proteins related to mitochondrial and neurological functions in the PFC was determined in each treatment group using proteomic and western blot analysis.

## Materials and Methods

### Experimental Design

**Figure 1:**
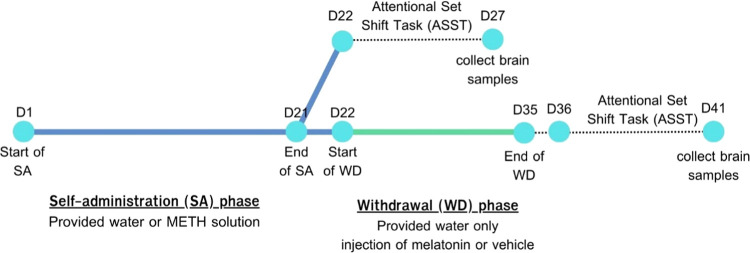
Experimental design and timelines for the self-administration (SA) phase, withdrawal (WD) phase, behavioral study, and brain sample collection.

Adult male ICR mice (6–8 weeks old, 20–30 g) were purchased from the National Laboratory Animal Center, Mahidol University, Thailand. All of the experimental procedures were performed according to the guidelines of the Laboratory Animal Care and Use Committee of Mahidol University (IMB-ACUC 2019/007 and 2023/003). Mice were individually housed on a 12 h light/12 h dark cycle at controlled temperature and humidity (temperature 22 ± 2 °C, relative humidity 55 + 15%) with ad libitum access to 2 drinking bottles and unlimited food. The weight of the mice was measured weekly, and their water consumption was measured daily. The mice were habituated in the animal facility for 1 week before the beginning of the experiment. The mice were divided into control and METH groups (*N* = 15/each group), and they drank various liquid contents in two bottles for 21 consecutive days, known as the self-administration phase (SA). In the SA phase, drinking bottle 1 of the METH group was filled with distilled water and drinking bottle 2 was filled with the solution of methamphetamine hydrochloride (Lipomed AG, AMP-732-HC-100, METH), which was dissolved in distilled water. The concentration of METH solution was adjusted weekly based on the body weight of the mice by using a concentration 0.05 mg/mL from days 1 to 9 and then 0.1 mg/mL from days 10 to 21. The control mice were provided with two bottles of distilled water. After 21 days of the SA phase, 5 mice from each group were randomly selected and evaluated for cognitive performance in an attentional set shift task (ASST) from days 22 to 26. The following day, they were anesthetized via isoflurane inhalation, followed by rapid decapitation to collect fresh brain samples. The remaining 10 mice of each group went through the withdrawal phase (WD), where the mice in both groups received only distilled water to drink for 14 days. During the WD phase, mice in each group were randomly divided into two subgroups (*N* = 5) and were subcutaneously injected with vehicle (2.5% ethanol in saline) or melatonin (10 mg/kg) (SIGMA-ALDRICH, M5250-1G) dissolved in vehicle once a day for 14 consecutive days. At the end of the WD phase, the mice were evaluated for their cognitive performance on days 36–40. The following day, they were decapitated for brain collection and subsequent protein analysis (Figure 1).

### Attentional Set Shift Task (ASST)

An attentional set shift task (ASST) was used to measure cognitive flexibility, self-inhibition, and working memory in mice. The mice completed the tasks as described in a previous experiment.^14^ In brief, the mice were put into an ASST box, divided into three chambers, one for a waiting area (30 × 30 cm^2^) and two for testing areas (15 × 15 cm^2^). The waiting chamber contained a water bowl and the testing chamber had a testing bowl. During the cognitive testing, each bowl contained different cues (medium and odor), with the mice learning which cues were relevant to a food reward while ignoring an irrelevant cue. The relevant cue was changed during the task to assess the cognitive flexibility of the mice. The mice had to perform a 7-stage task consisting of simple discrimination (SD), compound discrimination (CD), reversal discrimination 1 (RD1), intradimensional acquisition (ID), reversal discrimination 2 (RD2), extradimensional discrimination (ED), and reversal discrimination 3 (RD3). The criterion for completing each task was to correctly choose the positive bowl for 4 consecutive trials, with up to 20 trials available per task. The number of trials each mouse used to complete the task was recorded and measured as the number of trials per criterion.

### Sample Preparation for LC-MS/MS

For liquid chromatography with tandem mass spectrometry (LC-MS/MS), fresh brains were dissected and the whole PFC was collected from each mouse brain for proteomic analysis. Each sample was snap-frozen in liquid nitrogen and lyzed with ice-cold lysis buffer (30 mM Tris–HCl (pH 8.5), 7 M urea, 2 M thiourea, 4% CHAPS and 1% protease inhibitors cocktail). After that, the sample was centrifuged at 14,000*g* for 30 min at 4 °C. The supernatant was collected and cleaned up using a clean-up kit (GE Healthcare). The protein pellets were then dissolved in 8M urea and the total protein concentration of brain samples was evaluated using Bradford’s method (Bio-Rad protein assay, Bio-Rad Laboratory, CA). In the in-solution digestion process, the protein in each sample was digested into peptides. First, 50 μg of protein was reduced by incubating with a reduction buffer (100 mM dithiothreitol in 100 mM TEAB) for 30 min at room temperature. Then, alkylating buffer (100 mM iodoacetamide in 100 mM TEAB) was added to the sample, incubated for 30 min in the dark, and re-incubated with a reduction buffer for 15 min. The sample was digested into peptides with Trypsin Gold (mass spectrometry grade; Promega) for 16 h at 37 °C. The digested sample was dried in an evaporator, resuspended in 0.1% formic acid (FA), and cleaned by a C18 Zip tip. Finally, the concentration of cleaned peptides was measured by a NanoDrop 1000 spectrophotometer (Thermo Fisher Scientific, Bremen, Germany).

### Screening for Protein Profile Using Label-Free Nano-LC-MS/MS Analysis

The peptides were determined using an LC-MS/MS system (Dionex Ultimate 3000, RSLCnano System, Thermo Scientific) combined with CaptiveSpray source/Quadrupole ion trap mass spectrometry (model Q-ToF Compact, Bruker, Germany). Nano trap column 100 μm i.d. × 2 cm, Acclaim PepMap100 C18 5 μm, and pore size 100 Å were used for enriching and separating 1 μg of peptides. Then, the elution step was performed by using a linear gradient of 2–95% solvent B over 160 min at a flow rate of 300 nL/min and a PepMap100 C18 3 μm 75 μm × 500 mm LC column at 60 °C. The 0.1% FA in water and 0.08% FA in 80% acetonitrile were used as a mobile phase with the loading pump solvent consisting of 0.05% TFA in 2% acetonitrile. The concentration gradient of the mobile phase was used as 2, 35, 55, 95, and 2% by the time 5, 120, 20, 10 min and 1 s, respectively. The drying gas flow was 5 L/min at 150 °C, and the nebulizer gas pressure was 0.2 bars. The rate of MS acquisition was 6 Hz with a range of *m*/*z* as 150–2200 for mass scanning in positive ionization mode.

MaxQuant software and its built-in search engine, Andromeda, were used to analyze the screening phase’s MS data by blasting against the Mus musculus Uniprot database. A searching parameter was set, including fixed modification (carbamidomethyl cysteine) and variable modifications (N-terminal acetylation and methionine oxidation). The instrument type was Bruker Q-TOF with peptide tolerance of 0.5 and 0.25 for the first and main searches, respectively. The peptide identification was set as Trypsin/P with two maximum missing cleavages and a false discovery rate (FDR) of 1% of protein level. The time-of-flight (ToF) MS/MS match tolerance was set as 0.5 Da and the match between run option was selected. The software generated the label-free quantitation (LFQ) intensity of each matching protein, which was used for further statistical analysis.

### Quantification of the Expression of Proteins of Interest by SWATH-MS Analysis

The same samples as the screening phase were used in this step and conducted with the same machine and materials as in the screening phase but with a different protocol. The concentration gradient and elution time of mobile phase B were changed as follows: 2% at 0–5 min, 35% at 125 min, 55% at 145 min, 95% at 155 min, and 2% at 155–160 min. The MS machine was set up to SWATH mode, which changed the resolution of mass isolation width to 25 Da, which was set in a looped mode over 150–22,000 *m*/*z* and constructed 32 overlapping windows. Each ion fragment was set with an accumulation time of 100 ms and a total duty cycle of 3.0 s. The raw data from MS were analyzed by Skyline software (version 19.1.0.193) for generating a histogram of peptides. All peaks were manually checked and integrated into the total area sum (TAS) of peptide intensity. The TAS of all peptides composed of the same protein was used for calculating protein intensity. The mass spectrometry proteomics data have been deposited to the ProteomeXchange Consortium via the PRIDE partner repository with the dataset identifier PXD043828.

### Western Blot Analysis

The dissected PFC samples from each experimental group were lysed in RIPA buffer (50 mM Tris–HCl pH 7.4, 150 mM NaCl, 1 mM EDTA, 1% Triton X-100, 1% sodium deoxycholate, 0.1% SDS, and 1% protease inhibitor), then homogenized and centrifuged at 4 °C, 12,000*g* for 15 min. After that, the supernatant was collected to evaluate the protein concentration using the methods of Bradford (Bradford, 1976). Next, protein from each sample was mixed with the sample buffer (62.5 mM Tris–HCl, pH 6.8, 2% SDS, 10% glycerol, 2% mercaptoethanol, and 0.01% bromophenol blue) and denatured at 95 °C for 10 min. Then, the denatured samples, protein molecular weight marker (BlueEyed, PS-104, Jena Bioscience), and Precision Plus Protein Western C Blotting Standards (1610376, Bio-Rad) were loaded into SDS-PAGE gel and electrophoresed. Proteins separated by electrophoresis were transferred to poly(vinylidene difluoride) (PVDF) membranes (GE Healthcare Life Science). Then, PVDF membranes were blocked with 5% nonfat milk in Tris-buffered saline containing 0.1% Tween-20 (TBS-T) at room temperature for 1 h, followed by incubation with primary antibodies overnight at 4 °C. The primary antibodies included: mouse monoclonal anti-PINK1 (1:2000, sc-517353, Santa Cruz), mouse monoclonal anti-PARKIN1 (1:2000, 2132, Cell Signaling Technology), rabbit monoclonal anti-Synaptophysin (1:10000, 36406, Cell Signaling Technology), rabbit monoclonal anti-LC3B (1:10000, 2775, Cell Signaling Technology), and mouse monoclonal anti-actin (1:10000, 3700, Cell Signaling Technology). The following day, membranes were incubated with a horseradish-conjugated antirabbit (1:20000, 7074, Cell Signaling Technology) or antimouse IgG (1:5000–20000, 7076, Cell Signaling Technology) and 1 μg/10 mL StrepTactin HRP conjugate (1610380, Bio-Rad) for 60–90 min at room temperature. The signal of protein bands was enhanced with the ClarityWestern ECL Substrate (1705060, Bio-Rad) and detected using a Vilber Fusion FX7 Image analyzer. The density of immunoblot bands was analyzed by ImageJ software. All results were normalized to the expression of actin within the same sample.

### Statistical Analysis

The weekly mice weight and daily drinking consumption values from each group were analyzed by calculating the average across 21 days of the SA phase and 14 days of the WD phase and presented as mean ± SEM. In addition, the daily METH intake concentration was calculated using the following equation.
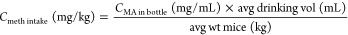
where *C*_meth intake_ is the meth intake concentration and *C*_MA in bottle_ is the MA concentration in the bottle.

Then, the significant change of drinking consumption and METH intake across 21 days was considered using a one-way analysis of variance (ANOVA), followed by Tukey’s correction for multiple comparison tests. The LC-MS/MS data was analyzed with Perseus software (version 1.6.8.0) for statistical calculation. All results from the METH group in the SA phase were analyzed by using an unpaired *t*-test to find a significant difference compared to the control group. The comparison of the results among groups during the WD phase was conducted using multiple comparisons with Tukey’s correction. A p-value of less than 0.05 determined the significant difference.

## Results

### Mouse Body Weight

The body weight of mice in control and METH groups was measured weekly across SA and WD periods. METH-consuming mice did not show a significantly different body weight compared to controls. In addition, the melatonin treatment did not affect the body weight of mice compared to the control group (Table 1).

**Table 1:** Average Body Weight and Total Weight Gain of Mice during the Self-Administration (SA) and Withdrawal (WD) Phases^a^

	SA phase		WD phase			
	control (*N* = 15)	METH (*N* = 15)	control (*N* = 5)	METH-WD (*N* = 5)	METH-WD/mel (*N* = 5)	mel (*N* = 5)
body weight (g) day 1	40.74 ± 5.11	36.85 ± 3.96	41.08 ± 3.48	37.14 ± 4.13	38.17 ± 3.90	40.3 ± 4.60
body weight (g) day 21	42.83 ± 3.25	39.42 ± 2.91				
body weight (g) day 35			42.51 ± 4.90	39.2 ± 3.46	39.75 ± 3.87	41.65 ± 3.79
total weight gain (g) day 1 vs day 21	2.09 ± 1.86	2.57 ± 1.05				
total weight gain (g) day 1 vs day 35			1.43 ± 1.48	2.06 ± 1.00	1.58 ± 0.66	1.35 ± 1.82

a The data express the mean ± SEM of the mice weight of the control and methamphetamine (METH) group from the self-administration (SA) phase and control, METH withdrawal (METH-WD), METH withdrawal with melatonin treatment (METH-WD/mel) and control with melatonin treatment (mel) during the withdrawal (WD) phase.

### Water Drinking Volume and METH Consumption Rate of Mice

**Figure 2:**
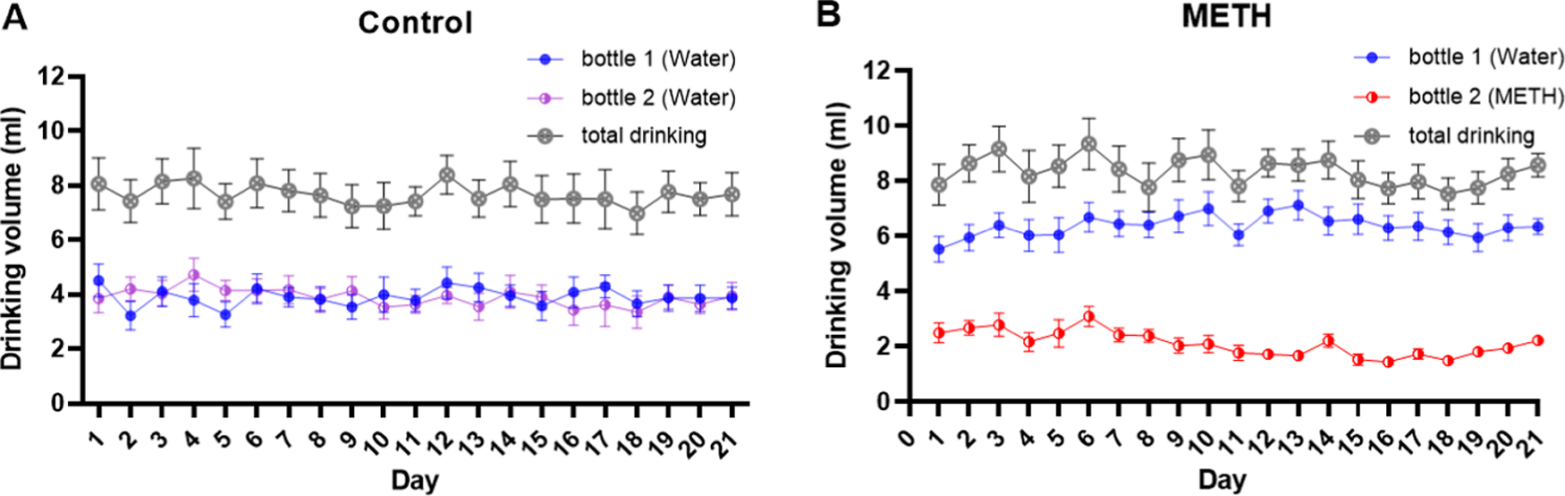
Drinking volume of mice during the self-administration (SA) phase for 21 consecutive days. (A) The drinking volume of water in bottles 1 and 2 of control mice and total volume. (B) The drinking volume of water in bottle 1, methamphetamine (METH) solution in bottle 2, and total volume. The data is represented as mean score ± SEM.

**Figure 3:**
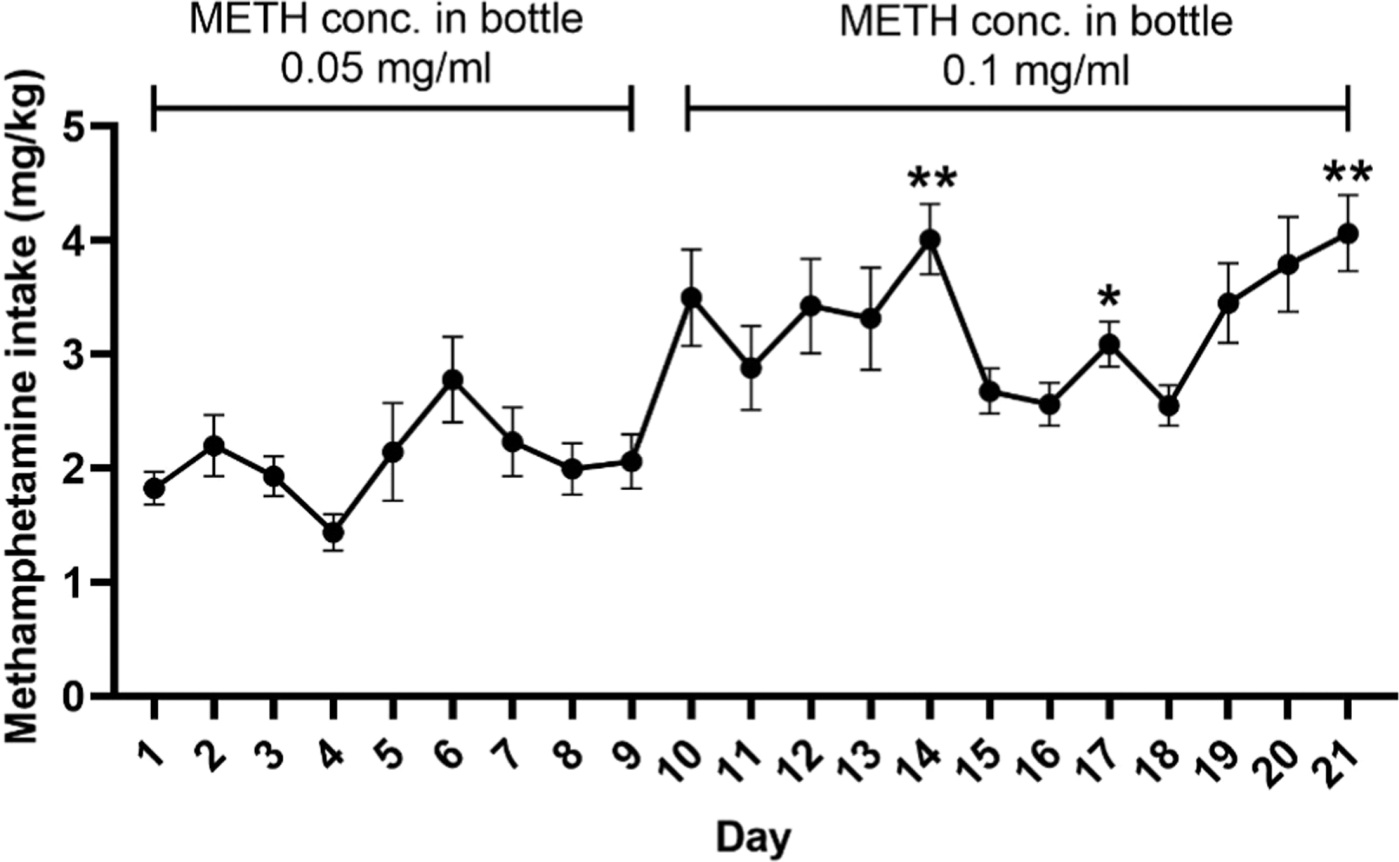
Concentration (conc.) of methamphetamine (METH) intake during self-administration (SA) for 21 consecutive days. The concentration of METH solution in bottles on days 1–9 and 10–21 was 0.05 and 0.1 mg/mL, respectively. The data is represented as mean score ± SEM and a significant change is noted as *P < 0.05 and **P < 0.01 compared to day 1.

The volume of water drinking in the SA phase was recorded daily for 21 days. The average total drinking volume of control and METH groups was 7.67 ± 0.37 mL (Figure 2A) and 8.35 ± 0.50 mL (Figure 2B), respectively, which did not show any significant differences between both groups. The data showed that the volume of METH solution consumed was less than the volume of water consumed in the METH group. However, the concentration of METH intake increased over time due to the concentration of METH solution in the bottle was adjusted by using 0.05 mg/mL on days 1–9 and changing to 0.1 mg/mL on days 10–21. Figure 3 indicates a slightly increased trend of METH intake concentration over time. The results showed a significant difference on days 14, 17, and 21 when compared to day 1, and the comparison between the other day is shown in the Supporting Information (Table S1). The average concentration of METH intake across 21 days was 2.67 ± 0.147 mg/kg/day (data not shown).

### Effect of METH Self-Administration, METH Withdwawal, and Melatonin Treatment on Cognitive Function of Mice

**Figure 4:**
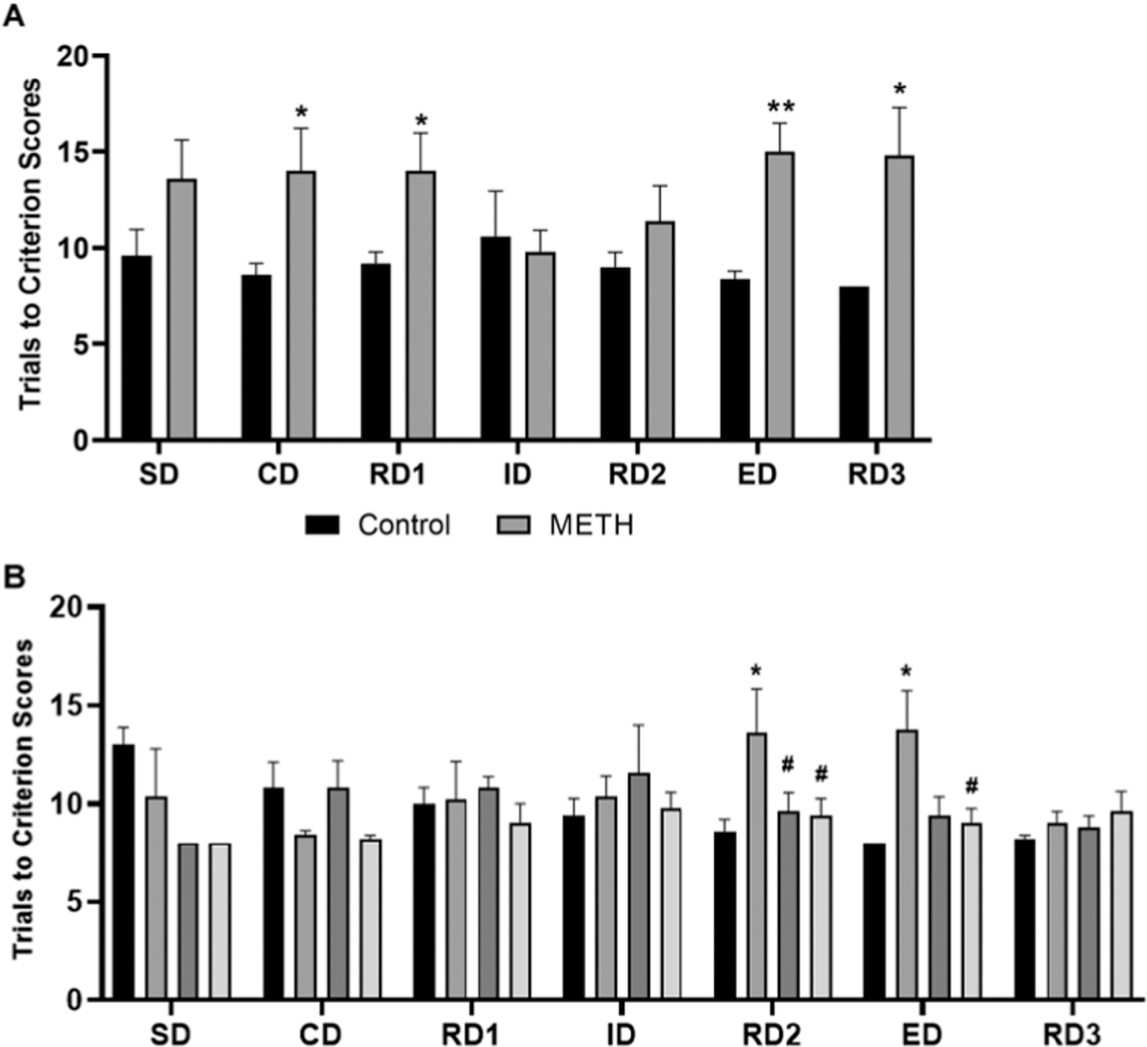
ASST scores of mice in different tasks. (A) The ASST score of control and methamphetamine (METH) intake mice in a self-administrated (SA) phase (*N* = 5/group). (B) The ASST score during the withdrawal (WD) period of control, METH withdrawal (METH-WD), METH withdrawal with melatonin injection (METH-WD/melatonin), and control with melatonin injection (mel) mice (*N* = 5/group). The data is represented as mean score ± SEM and significant change is noted as *P < 0.05 and **P < 0.01 compared to control mice, and ^#^*P* < 0.05 compared to METH mice.

After the SA phase, METH administration for 21 days significantly increased Trials to Criterion scores of the ASST test of METH mice in task CD (5.40 ± 2.29, *P* < 0.05), RD1 (4.80 ± 2.06, *P* < 0.05), ED (6.60 ± 1.54, *P* < 0.01), and RD3 (6.80 ± 2.50, *P* < 0.05) when compared with the control group (Figure 4A). After 14 days of the WD phase, the Trials to Criterion scores of the ASST test were significantly increased in RD2 (5.80 ± 1.66) and remained increased in the ED (5.80 ± 1.64) of the METH group when compared to the control group. Melatonin treatment during the WD phase reversed the METH-induced increase in the Trials to Criterion scores of the ASST test of RD2. In contrast, melatonin treatment did not cause any significant change in the ASST scores in the water control group (control compared to mel, Figure 4B).

### Screening the Protein Profile of the Prefrontal Cortex of Mice at METH Self-Administration or Withdrawal Phase

**Figure 5:**
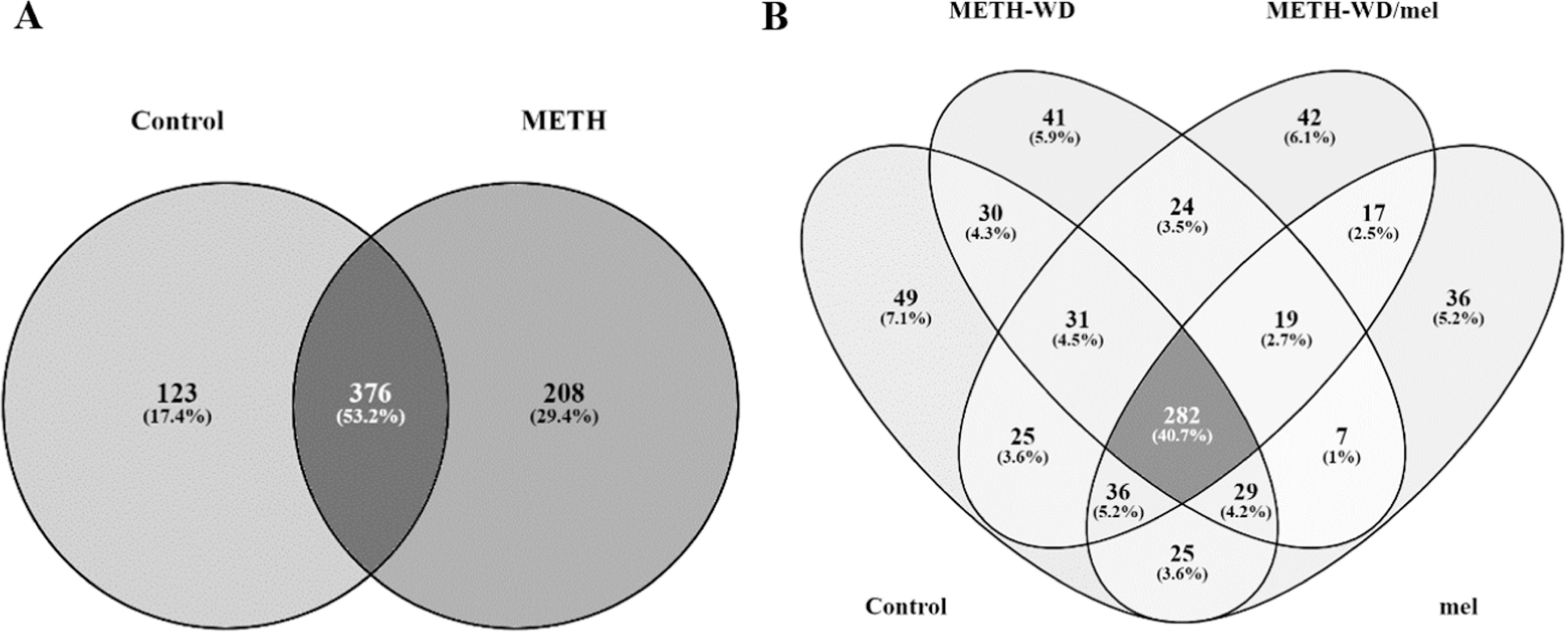
Venn diagram illustrating the number of identified proteins using label-free Nano-LC Q-TOF analysis. (A) The diagram of the number of discovered proteins in the self-administration (SA) phase from control and methamphetamine (METH) groups. (B) The diagram of the number of discovered proteins in the withdrawal (WD) phase of control, METH withdrawal (METH-WD), METH withdrawal with melatonin treatment (METH-WD/mel), and control with melatonin treatment (mel) groups.

**Figure 6:**
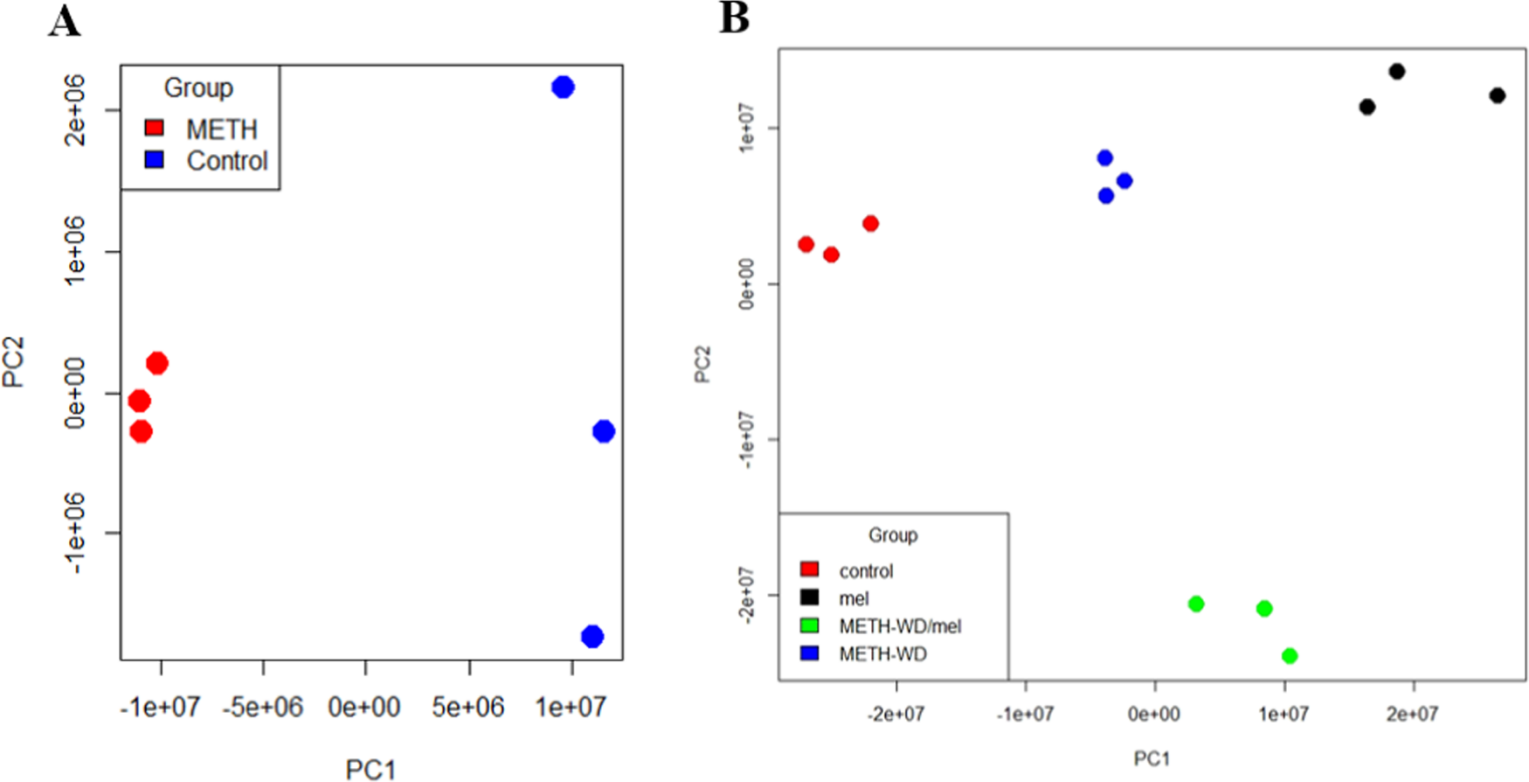
Principal component analysis (PCA) of LFQ intensity of proteins from individual samples. (A) PCA diagram of brain samples in the self-administration period comprised of control (blue) and METH (red) samples. (B) PCA diagram of brain samples in the withdrawal (WD) period comprised of control (red), METH withdrawal (METH-WD) (blue), METH withdrawal with melatonin treatment (METH-WD/mel) (green), and control with melatonin treatment (mel) (black) samples. These diagrams were calculated and visualized by R software.

Protein levels from the PFC of individual mice in each group were quantified by nontargeted label-free Nano-LC quadrupole time-of-flight (Q-TOF) analysis, generating their protein profile together with LFQ values. The software identified 941 proteins in control and METH mice samples from the SA phase, with 707 proteins remaining after nonspecific proteins were excluded. The Venn diagram shows that the number of unique proteins in control and METH mice was 123 and 208, respectively, and 376 proteins were shared in both groups (Figure 5A). The principal component analysis (PCA) diagram revealed in-group variation of protein expression and discriminated differences in expression between groups. The results illustrate the difference between the protein expression of METH-treated mice and control in different areas of the diagram. METH treatment affected the global protein expression levels of PCF as assessed by the PCA (Figure 6). Mice treated with METH showed a compact distribution of protein expression, while the control showed a wider distribution (Figure 6A).

The results of samples from the WD phase showed that 888 proteins were identified, and 693 proteins were determined after eliminating nonspecific proteins. The total number of proteins found in control, control with melatonin treatment (mel), METH withdrawal (METH-WD), and METH withdrawal with melatonin treatment (METH-WD/mel) groups were 507, 451, 463, and 476, respectively (Figure 5B). The Venn diagram shows that 282 proteins were shared among the four treatment groups, and 7–49 proteins were shared between two or three treatment groups. The PCA diagram shows noticeable protein expression differences among all groups with high in-group consistency (Figure 6B).

**Figure 7:**
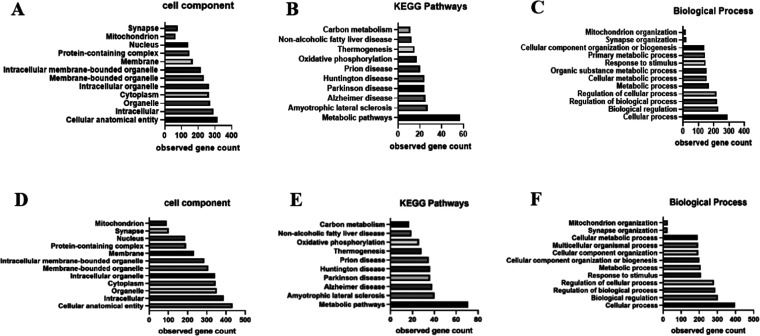
Classification of significantly changing protein expression due to GO of cellular components, KEGG pathway, and biological process. (A–C) The observed gene count of proteins from self-administration period samples. (D–F) The observed gene count of proteins from withdrawal period samples.

The LFQ intensity of each protein in the PFC demonstrated that during the SA phase, 372 proteins significantly changed (*p* < 0.001) when compared between the control and METH groups (Table S2). The results from the WD phase showed a significant change of 498 proteins (*P* < 0.001) among 4 groups with ANOVA comparison (Table S3). The 372 proteins in the SA phase and the 498 proteins in the WD phase were classified by Gene ontology (GO) terms into their cellular components, KEGG pathway, and biological process (Figure 7). Of these, 16 proteins were then selected from the GO terms based on their function in the mitochondrion, mitochondrial organization, synaptic formation, and neurological disorders. These proteins were determined as proteins of interest, which were qualified in their expression by SWATH analysis.

### Quantification of the Expression of Proteins of Interest by SWATH-MS and Western Blot Analysis from the Prefrontal Cortex of Mice at METH Self-Administration or Withdrawal Phase

The 16 proteins of interest from the screening phase involve mitochondrial organization, synaptic formation, and oxidation phosphorylation (OXPHOS) pathway. The expression of these proteins was quantified by targeted label-free Nano-LC Q-ToF with SWATH-MS mode. Then, the raw data was processed by Skyline software and generated total area sum (TAS) of each protein of interest. The expression of proteins was calculated by the ratio of METH/control in the log 2 scale. The log 2 scale values above 1 or below −1 represent an increase or decrease in protein intensity over 2-fold in the METH group compared to the control group (Table 2).

**Table 2:** Comparison of the Protein Intensity from SWATH-MS Analysis during the SA Phase between METH and Control Groups^a^

no.	gene name	protein name	log 2 ratio of protein intensity (METH/control)	biological process	KEGG pathway	cellular components
1	Opa1	dynamin-like 120 kDa protein, mitochondrial	1.41^#^	mitochondrial organization	n/a	mitochondrion
2	Park7	Parkinson’s disease protein 7	1.34	mitochondrial organization	Parkinson’s disease	mitochondrion Synapse
3	Uqcrc2	cytochrome b-c1 complex subunit 2, mitochondrial	–0.99	mitochondrial organization	Parkinson’s disease Alzheimer’s disease	mitochondrion
4	Syn1	synapsin-1	–1.55	regulation of synaptic plasticity	n/a	synapse
5	Syp	synaptophysin	–1.71	regulation of synaptic plasticity	n/a	synapse
6	Stxbp1	syntaxin-binding protein 1	–1.31^#^	regulation of synaptic plasticity	synaptic vesicle cycle	mitochondrion Synapse
7	Vps35	vacuolar protein sorting-associated protein 35	0.98	synapse organization	n/a	mitochondrion Synapse
				Mitochondrial organization		
8	Vamp2	vesicle-associated membrane protein 2	1.30	regulation of synaptic plasticity	synaptic vesicle cycle	synapse
9	Rab3a	ras-related protein Rab-3A	1.03	regulation of synaptic plasticity mitochondrial organization	synaptic vesicle cycle	synapse
10	Mapt	microtubule-associated protein tau	1.40	regulation of synaptic plasticity mitochondrial organization	Parkinson’s disease Alzheimer’s disease	synapse
11	Snca	α-synuclein	3.04	OXPHOS	Parkinson’s disease	mitochondrion synapse
				regulation of synaptic plasticity mitochondrial organization		
12	Ndufs6	NADH dehydrogenase iron–sulfur protein 6, mitochondrial	–1.60^#^	OXPHOS	Parkinson’s disease Alzheimer’s disease	mitochondrion
				mitochondrial organization		
13	Ndufb4	NADH dehydrogenase 1 β subcomplex subunit 4	–1.27	OXPHOS	Parkinson’s disease Alzheimer’s disease	mitochondrion
				mitochondrial organization		
14	Sdhb	succinate dehydrogenase iron–sulfur subunit, mitochondrial	–1.26	OXPHOS	Parkinson’s disease Alzheimer’s disease	mitochondrion
15	Uqcrb	cytochrome b-c1 complex subunit 7	–1.56^#^	OXPHOS	Parkinson’s disease Alzheimer’s disease	mitochondrion
				mitochondrial organization		
16	Cox6c	cytochrome c oxidase subunit 6C	–2.10	OXPHOS	Parkinson’s disease Alzheimer’s disease	mitochondrion

a The data express the log 2 ratio of protein expression of methamphetamine (METH) by control groups. *P*-values were calculated by unpaired *t*-test and the significant change was shown as ^#^*P* < 0.05 when comparing METH to control.

The SA phase results illustrate a significant change in several proteins involved with mitochondrial organization, biogenesis, and synaptic plasticity when compared between METH and control groups. Dynamin-like 129 kDa (OPA1) expression was significantly increased (*P* < 0.05) in the METH group. In contrast, syntaxin-binding protein 1 (Stxbp1), NADH dehydrogenase iron–sulfur protein 6, mitochondrial (Ndufs6), and cytochrome b-c1 complex subunit 7 (Uqcrb) were significantly decreased (*P* < 0.05) due to METH treatment. Additionally, some mitochondrial-related proteins did not reach statistical significance but showed a high change in their expression, more than 2-fold when compared between METH and control. For instance, the proteins that regulate mitochondrial dynamic and synaptic function were shown to have a 2-fold increase in the METH group, including Parkinson’s disease protein 7 (Park7), vacuolar protein sorting-associated protein 35 (Vps35), vesicle-associated membrane protein 2 (Vamp2), Ras-related protein Rab-3A (Rab3a), and microtubule-associated protein tau (Mapt). In addition, several proteins, which play an important role in synaptic function and OXPHOS pathway, were shown to have a nonsignificant yet 2-fold decrease in the METH group, such as synapsin-1 (Syn1), synaptophysin (Syp), NADH dehydrogenase 1 β subcomplex subunit 4 (Ndufb4), succinate dehydrogenase iron–sulfur subunit, mitochondrial (Sdhb), and cytochrome c oxidase subunit 6C (Cox6c). However, most of these protein changes recovered to control values after 14 days of the WD phase except Syp, which decreased significantly (*P* < 0.05) in METH-WD compared to the control. Furthermore, melatonin treatment causes a change in the expression of proteins that are involved with GO terms concerning mitochondrial organization, synaptic formation, or OXPHOS pathway when compared to control and METH-WD mice with saline injection. The following proteins that show a 2-fold decreasing trend were Park7, Vps35, Snca, and Mtco2. In contrast, some proteins expressed at low levels in METH-WD mice showed an increasing trend after melatonin treatment, for instance, Syn1, Syp, Ndufb4, and Cox6c. Moreover, the expression of some proteins in the melatonin-treated group compared to controls was significantly increased by melatonin treatment, such as Uqcrc2 and Ndufs6 (Table 3).

**Table 3:** Protein Intensity from SWATH-MS Analysis during the Withdrawal Phase was Compared among the Four Treatment Groups^a^

			log 2 ratio of protein intensity						
no.	gene name	protein name	M/C	Mm/C	m/C	Mm/M	biological process	KEGG pathway	cellular components
1	Opa1	dynamin-like 120 kDa protein, mitochondrial	–0.185	–0.406	–0.999	–0.221	mitochondrial organization	n/a	mitochondrion
2	Park7	Parkinson’s disease protein 7	0.108	–1.148^#^	–0.980	–1.256*	mitochondrial organization	Parkinson’s disease	mitochondrion synapse
3	Uqcrc2	cytochrome b-c1 complex subunit 2, mitochondrial	0.429	–0.234	1.014	–0.662	mitochondrial organization	Parkinson’s disease Alzheimer’s disease	mitochondrion
4	Syn1	cynapsin-1	0.201	1.267^#^	0.050	1.065*	regulation of synaptic plasticity	n/a	synapse
5	Syp	cynaptophysin	–1.722^#^	–0.598	0.464	1.123	regulation of synaptic plasticity	n/a	synapse
6	Stxbp1	cyntaxin-binding protein 1	–0.014	–0.910	–0.053	–0.896	regulation of synaptic plasticity	synaptic vesicle cycle	mitochondrion synapse
7	Vps35	vacuolar protein sorting-associated protein 35	0.236	–1.067	–0.681	–1.303	synapse organization	n/a	mitochondrion synapse
							mitochondrial organization		
8	Vamp2	vesicle-associated membrane protein 2	0.133	–0.853	0.427	–0.985	regulation of synaptic plasticity	synaptic vesicle cycle	synapse
9	Rab3a	ras-related protein Rab-3A	0.658	–0.079	0.465	–0.736	regulation of synaptic plasticity mitochondrial organization	synaptic vesicle cycle	synapse
10	Mapt	microtubule-associated protein tau	0.528	–0.087	0.177	–0.615	regulation of synaptic plasticity mitochondrial organization	Parkinson’s disease Alzheimer’s disease	synapse
11	Snca	α-synuclein	–0.645	–1.531^#^	–0.096	–0.885	OXPHOS regulation of synaptic plasticity mitochondrial organization	Parkinson’s disease	mitochondrion synapse
12	Ndufs6	NADH dehydrogenase iron–sulfur protein 6, mitochondrial	0.040	–0.628	2.104	–0.668	OXPHOS	Parkinson’s disease Alzheimer’s disease	mitochondrion
							mitochondrial organization		
13	Ndufb4	NADH dehydrogenase 1 β subcomplex subunit 4	–0.411	1.231	–0.737	1.642	OXPHOS	Parkinson’s disease Alzheimer’s disease	mitochondrion
							mitochondrial organization		
14	Sdhb	succinate dehydrogenase iron–sulfur subunit, mitochondrial	0.386	0.532	0.171	0.145	OXPHOS	Parkinson’s disease Alzheimer’s disease	mitochondrion
15	Uqcrb	cytochrome b-c1 complex subunit 7	0.683	0.477	0.674	–0.206	OXPHOS	Parkinson’s disease Alzheimer’s disease	mitochondrion
							mitochondrial organization		
16	Cox6c	cytochrome c oxidase subunit 6C	–0.325	1.692	–0.606	2.017	OXPHOS	Parkinson’s disease Alzheimer’s disease	mitochondrion

a The data express the log2 ratio of protein expression of methamphetamine withdrawal (METH-WD) by control (M/C), METH-WD with melatonin injection (METH-WD/mel) by control (Mm/C), control with melatonin injection (mel) by control (m/C), and METH-WD/mel by METH-WD (Mm/M), respectively. The significant difference was calculated by Tukey’s multiple comparison tests and the significant change was shown as ^**#**^*P* < 0.05 compared to the control group and *P < 0.05 compared to the METH group.

**Figure 8:**
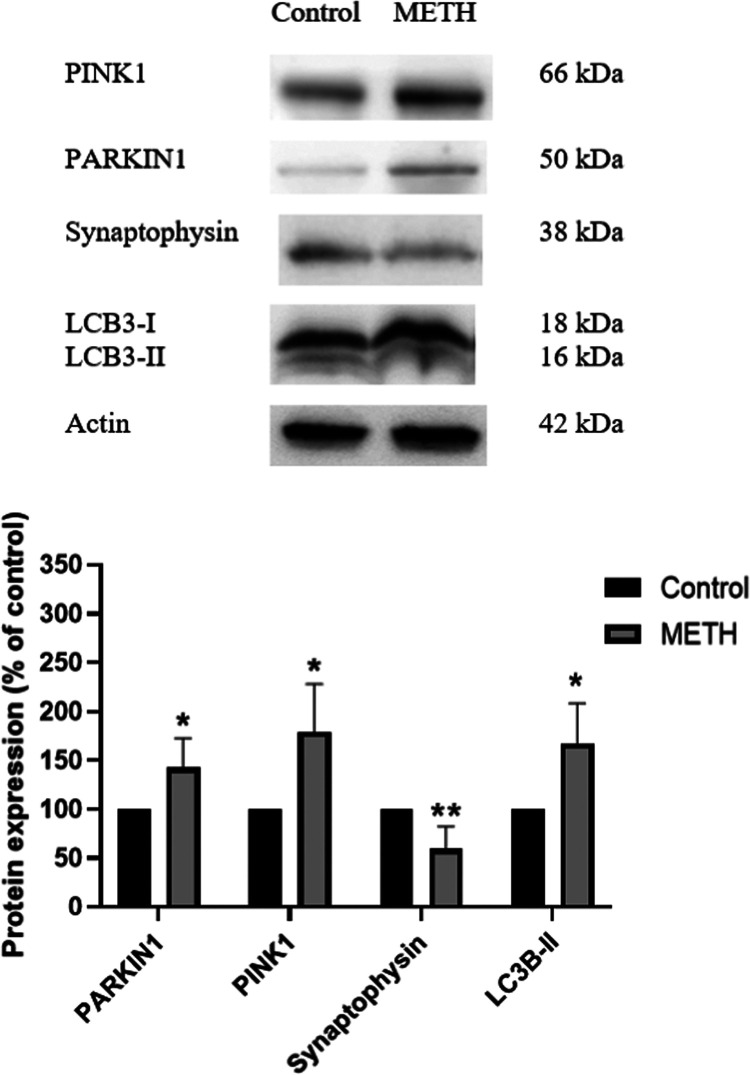
Western blot analysis of mitophagy and synaptic markers on the prefrontal cortex (PFC) of mice in each group from the self-administration phase (*N* = 3/group). The data are expressed as mean + SEM and a significant difference is noted as *P < 0.05 and **P < 0.01 compared to the control group. Full membrane picture is shown in the Supporting Information (Figure S1).

**Figure 9:**
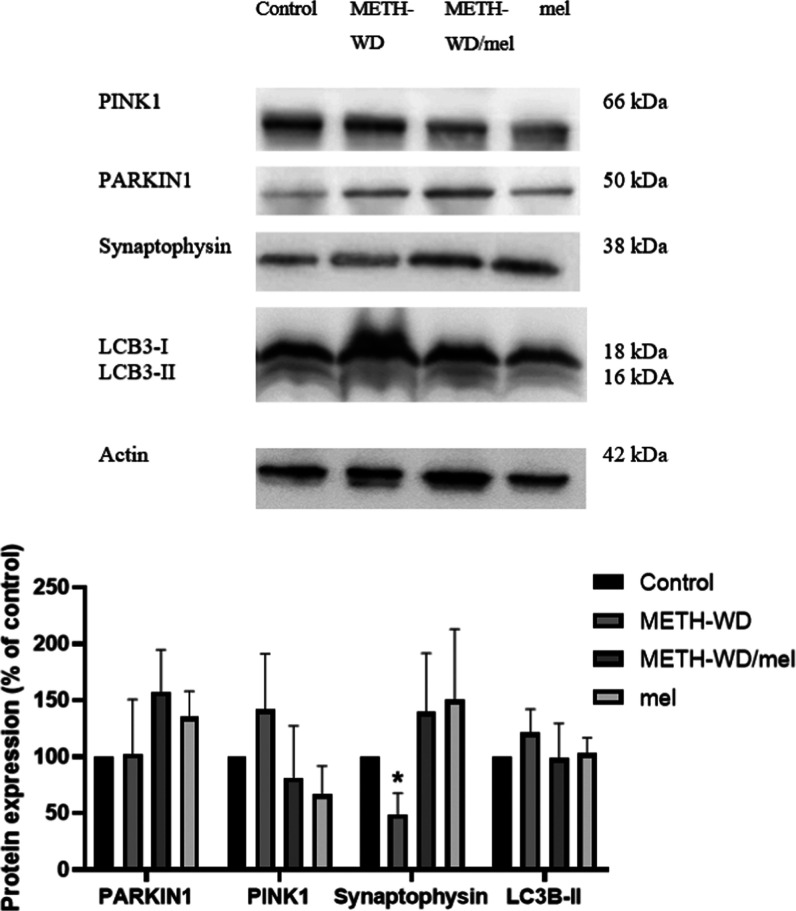
Western blot analysis of mitophagy and synaptic markers on the prefrontal cortex (PFC) of control, METH withdrawal (METH-WD), METH withdrawal with melatonin treatment (METH-WD/mel), and control with melatonin treatment (mel) groups from the withdrawal (WD) phase (*N* = 3/group). The data are expressed as mean + SEM and a significant difference is noted as *P < 0.05 compared to the control. Full membrane picture is shown in the Supporting Information (Figure S1).

According to label-free and SWATH analysis, the results showed the effect of METH treatment on the expression of proteins related to the regulation of mitochondrial function that may induce cognitive dysfunction or cell death. Western blot analysis was then used to confirm possible proteins and related pathways of METH-induced mitochondrial dysfunction, which may lead to autophagy-dependent cell death via excessive mitophagy induction. The results showed that mitophagy-related markers, such as E3 ubiquitin-protein ligase parkin (Parkin), PTEN-induced kinase 1 (PINK1), and microtubule-associated protein light chain 3 (LC3), were upregulated during the SA phase (Figure 8), and they returned to control levels after the WD phase (Figure 9). However, the synaptic-related protein, synaptophysin, was significantly decreased in the METH group compared to the control. Moreover, a significant decrease in synaptophysin was still observed in the METH-WD group after 14 days of the WD phase. Furthermore, the results from Western blot analysis indicate a therapeutic effect of melatonin treatment to regulate mitophagy-related proteins, showing a normalized expression level of Parkin, PINK1, and LC3 in the brain of METH-WD/mel and an increasing trend of synaptophysin expression in METH-WD/mel and mel mice when compared to METH-WD and control groups.

## Discussion

The present study revealed that chronic oral METH intake impaired cognitive flexibility measured by the ASST in mice. The ASST results during the self-administration (SA) phase demonstrate higher scores, indicating less flexibility, of METH-SA mice than controls, especially in SD, CD, ED, and RD3 tasks. The ASSTs are mainly used for evaluating cognitive flexibility in the domains of working memory, cognitive attention, and self-inhibition.^14^ Therefore, the METH mice showed poorer performance in these tasks, representing impairments in these areas of executive function. Interestingly, the behavioral results are related to a change in the proteomics profile of PFC in mice, with METH-treated mice, displaying changes in proteins involved in synaptic formation and neurotransmitter release. The proteomic results of the PFC from the SA phase illustrated decreased levels of syntaxin-binding protein 1 (Stxbp1), synapsin I (Syn1), and synaptophysin (Syp). These proteins are mainly present in presynaptic neurons and play an important role in synaptogenesis and presynaptic vesicle secretion.^15−17^ Previous research has found that a decrease in these proteins can cause the collapse of synaptic formation, leading to the apoptosis of neurons.^18,19^ Moreover, the present result reveals the effect of METH during SA for upregulating α-synuclein (Snca) and microtubule-associated protein tau (Mapt), which leads to neurodegeneration and synaptic toxicity.^20,21^ We also found that many proteins, which are synaptic vesicle-recycling machinery components, are overexpressed in the METH group when compared to the control group and may indicate compensatory stress response mechanisms. For example, the increase of Vps35 and Rab3a could indicate a response to combat α-synuclein accumulation and modulation of BACE1 activity.^22−24^ The increase in Vamp2 may occur to maintain axonal membrane fusion and the neurotransmitter-releasing process.^25,26^ The change of synaptic machinery at presynaptic sites might be caused by a defect in mitochondrial function, which plays a crucial role in supporting synaptic function and neurotransmitter release.^27^ METH-induced decrease of the proteins Ndufs6, Ndufb4, Sdhb, Uqcrc2, and Cox6c, which are components of the electron transport chain (Complex I–IV), might suggest mitochondrial dysfunction that regulates mitochondrial bioenergetics of the cell. Changes to these proteins may indicate impairment of the cell’s adenosine triphosphate (ATP) production due to METH-SA, resulting in synaptic dysfunction and cell death.^9^ The results also showed increased mitochondrial quality control proteins, such as OPA1 and Park7, which could promote mitochondrial hyperfusion and mitophagy as a stress response process to compensate against METH-induced neurotoxicity.^28,29^ To determine if these results are in relation to METH-induced toxicity to disturb mitochondrial quality control, we used SWATH analysis to find downstream mechanisms that might link METH-induced mitochondrial dysregulation and autophagic cell death.

Interestingly, SWATH and western blot analysis showed increased PARKIN, PINK, and LC3 protein levels in METH-SA mice when compared to controls. These proteins are involved in the initiation of mitophagy and autophagy processes, which could promote cell survival.^30^ This finding might indicate that chronic METH exposure activates mitophagy processes. However, excessive mitophagy activation could also accelerate mitochondrial destruction, which leads to a sudden decrease in energy production. This process could lead to autophagy-dependent cell death in the brain,^11,31^ causing cognitive impairment in the mice at the end of the SA period. However, the cognitive impairment in METH-SA mice (day 21) partly recovered during the METH withdrawal (WD) phase, as can be seen from the ASST result of METH mice after 14 days of METH-WD (day 35), which shows a recovery of some ASSTs except the RD2 and ED tasks. The recovery of cognitive function during the WD phase correlated with the restoration of proteins involved in mitochondrial function and autophagic cell death. Therefore, the current study has demonstrated that chronic METH administration caused the impairment of synaptic processes that may induce cognitive deficits. Moreover, the cognitive deficit may partially recover after METH withdrawal. Our behavioral and proteomic results of METH-induced neurotoxicity support previous research, where there is restoration of cognitive function in long-term METH abstinence patients.^32^

During the WD phase, the treatment with melatonin, a neuroprotective compound, diminished the enduring cognitive and proteomic changes induced by METH-SA, as shown by recovery of ASSTs and return of synapsin I expression to control levels. However, the treatment with melatonin in the control groups did not impact baseline cognitive performance. Interestingly, melatonin treatment promoted the expression of synaptic proteins, such as Syn1 and Syp, and modulated the expression of Snca and Mapt, which may help stabilize synaptic function and induce neuronal plasticity.^33^ Furthermore, melatonin treatment may regulate the bioenergetic process of the mitochondrion by promoting protein expression in the OXPHOS complex, such as Ndufs6, Ndufb4, and Cox6c, and support previous research that indicates an effect of melatonin to improve the activity of electron transport chain in complex I–IV.^34^ Melatonin treatment also modulated the mitochondrial dynamic and quality control process induced by METH. The results of melatonin treatment on restoration in the OPA, PARKIN, PINK1, and LC3 1 protein and downregulation of Park7, a gene encoded by DJ-1 in the METH-WD phase, might indicate a potential role of melatonin to modulate the mitophagy process. While the modulation mechanism remains unclear, there is evidence that melatonin could modulate the DJ-1/KLF17/ID-1 signaling pathway to prevent the excessive mitotic activities, as shown with the occurrence of cancer.^35^

In conclusion, in this study, the neurological assessment of cognitive function and proteomic analysis demonstrates that METH-induced mitochondrial dysfunction due to dysregulation of mitochondrial dynamic and bioenergetic production leads to autophagy-dependent cell death. Moreover, synaptic plasticity is more vulnerable to toxicity induced by METH than mitochondrial dysfunction and autophagy-dependent cell death. Taken together, the impairment in synaptic function might suggest to play important roles and be involved in cognitive deficits. Future studies to identify electrophysiological, molecular, cell biological, and proteomic approaches will provide more meaningful insights into the molecular mechanisms of synaptic dysfunction. Furthermore, our finding suggests the benefit of melatonin treatment in enhancing the recovery process from cognitive impairment caused by chronic METH administration.

## Supporting Information Available

Layout of western blot membrane and entire membrane with protein bands (Figure S1); post-hoc multiple comparison of METH intake concentration across 21 days of self-administration phase (Table S1) (PDF)
The protein list and intensity of self-administration phase (SD) brain samples from label-free LC-MS/MS analysis (Table S2) (XLSX)
The protein list and intensity of withdrawal phase (WD) brain samples from label-free LC-MS/MS analysis (Table S3) (XLSX)
