# Supplementary material for: Proteomic Analysis Reveals the Neurotoxic Effects of Chronic Methamphetamine Self-Administration-Induced Cognitive Impairments and the Role of Melatonin-Enhanced Restorative Process during Methamphetamine Withdrawal
Source: J Proteome Res. 2023 Sep 7;22(10):3348–59. doi: 10.1021/acs.jproteome.3c00502 (PMC10563163; doi:10.1021/acs.jproteome.3c00502)
Supplement: Supplementary file 1 — pr3c00502_si_001.pdf [file pr3c00502_si_001.pdf]

Supporting information

Proteomic analysis reveals the neurotoxic effects of chronic self-administration of methamphetamine-induced cognitive impairment and the restorative effect of melatonin after drug withdrawal.

Tanthai Polvat [1,3], Tanya Prasertporn [1], Piyada Na Nakorn [2], Supitcha Pannengpetch [2], Wilasinee Suwanjang [2], Jiraporn Panmanee [1], Sukhonthar Ngampramuan [1], Jennifer L. Cornish [3], Banthit Chetsawang [1]*

[1] *Research Center for Neuroscience, Institute of Molecular Biosciences, Mahidol University, Salaya, Nakhon Pathom, 73170, Thailand.*

[2] *Center for Research Innovation and Bioinformatics, Faculty of Medical Technology, Mahidol University, Salaya, Nakhon Pathom,73170, Thailand.*

[3] *Center of emotional health, Department of Psychology, Macquarie University, Balaclava Road, North Ryde, NSW, 2109, Australia.*

Table of content

Figure S1: The layout of the western blot membrane and the entire membrane with protein bands

Table S1: Post-hoc multiple comparisons of METH intake concentration across 21 days of self-administration phase

Table S2: The protein list and intensity from label-free LC-MS/MS analysis of self-administrated (SA) phase brain samples

Table S3: The protein list and intensity from label-free LC-MS/MS analysis of withdrawal phase (WD) brain samples

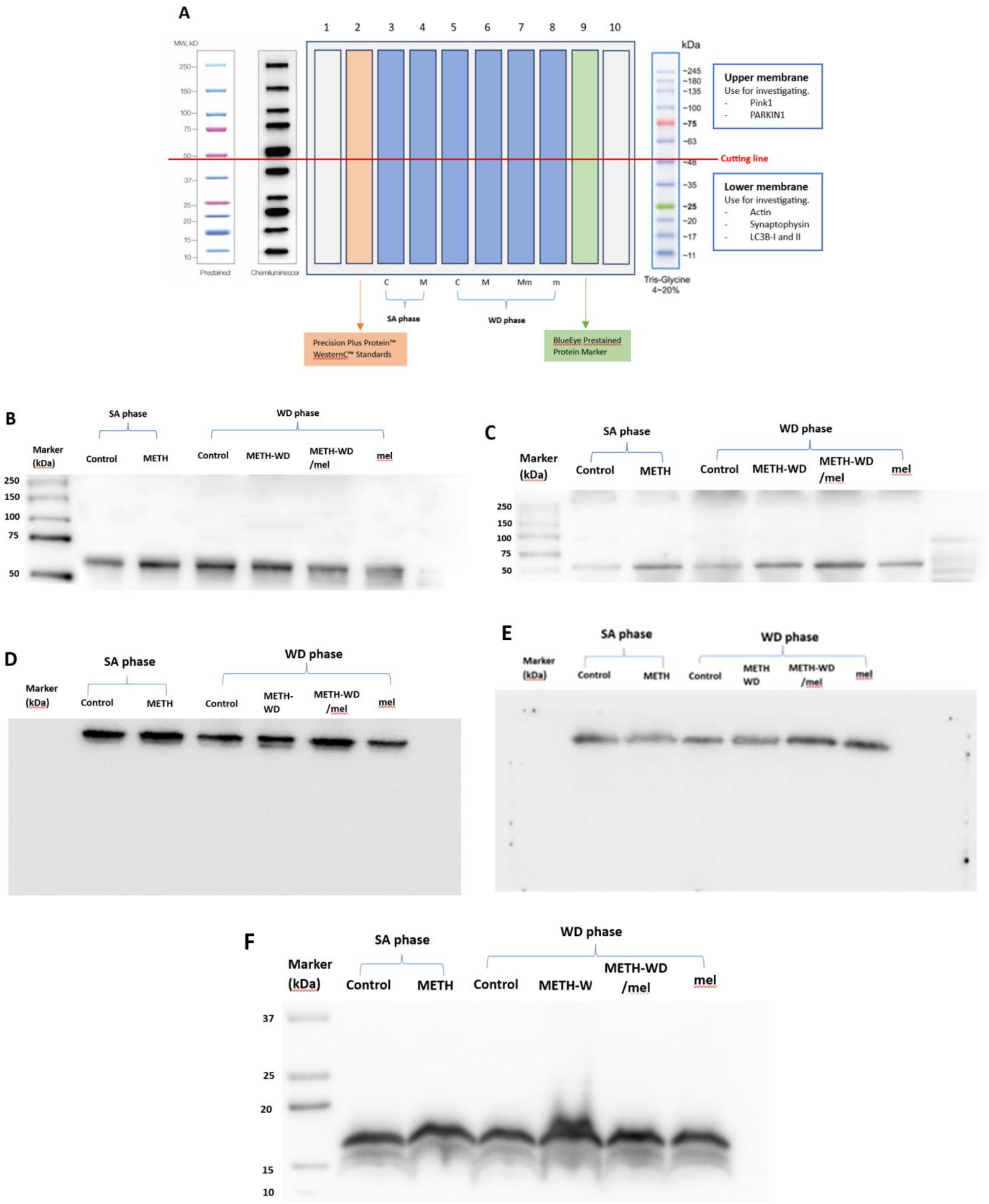

Figure S1 **(A)** Layout of western blot membrane for loading samples from experimental groups, control (C), METH (M), METH treated with melatonin (Mm) and control treated with melatonin (m) of self-administration phase (SA) and withdrawal phase (WD). **(B-F)** Entire membranes of

western blot analysis showed a band of PINK1(66kDa), PARKIN1 (50 kDa), Actin (42kDa), Synaptophysin (38 kDa), LC3B-I and LC3B-II (18,16 kDa), respectively.

Table S1 Post-hoc multiple comparisons of METH intake concentration across 21 days of self-administration phase

Multiple comparisons test (Day vs. Day)	Mean difference	95% Confidential Interval of difference	Significant	Adjusted P-value
**1 vs. 2**	-0.3737	-1.635 to 0.8876	ns	0.9967
**1 vs. 3**	-0.1051	-0.7782 to 0.5680	ns	>0.9999
**1 vs. 4**	0.3874	-0.4541 to 1.229	ns	0.8067
**1 vs. 5**	-0.3183	-2.452 to 1.815	ns	>0.9999
**1 vs. 6**	-0.9525	-2.880 to 0.9752	ns	0.8008
**1 vs. 7**	-0.4076	-2.030 to 1.215	ns	0.9995
**1 vs. 8**	-0.1672	-1.404 to 1.070	ns	>0.9999
**1 vs. 9**	-0.2334	-1.620 to 1.153	ns	>0.9999
**1 vs. 10**	-1.674	-3.410 to 0.06222	ns	0.0634
**1 vs. 11**	-1.055	-2.764 to 0.6544	ns	0.4945
**1 vs. 12**	-1.599	-3.767 to 0.5694	ns	0.2741
**1 vs. 13**	-1.49	-3.421 to 0.4417	ns	0.2159
**1 vs. 14**	-2.186	-3.916 to -0.4559	**	0.0082
**1 vs. 15**	-0.8525	-1.953 to 0.2476	ns	0.2113
**1 vs. 16**	-0.7365	-1.713 to 0.2404	ns	0.2488
**1 vs. 17**	-1.263	-2.419 to -0.1070	*	0.0271
**1 vs. 18**	-0.7245	-1.895 to 0.4457	ns	0.49
**1 vs. 19**	-1.625	-3.605 to 0.3546	ns	0.1648
**1 vs. 20**	-1.963	-4.147 to 0.2204	ns	0.099
**1 vs. 21**	-2.238	-3.867 to -0.6099	**	0.0039
**2 vs. 3**	0.2685	-1.059 to 1.596	ns	>0.9999
**2 vs. 4**	0.7611	-0.1320 to 1.654	ns	0.1269
**2 vs. 5**	0.05531	-1.042 to 1.153	ns	>0.9999
**2 vs. 6**	-0.5788	-2.320 to 1.162	ns	0.9913
**2 vs. 7**	-0.03392	-1.525 to 1.457	ns	>0.9999
**2 vs. 8**	0.2064	-1.047 to 1.460	ns	>0.9999
**2 vs. 9**	0.1403	-1.091 to 1.372	ns	>0.9999
**2 vs. 10**	-1.3	-3.639 to 1.039	ns	0.6604
**2 vs. 11**	-0.6812	-2.500 to 1.138	ns	0.969
**2 vs. 12**	-1.225	-2.577 to 0.1266	ns	0.096
**2 vs. 13**	-1.116	-2.129 to -0.1028	*	0.0255
**2 vs. 14**	-1.812	-4.085 to 0.4606	ns	0.1972
**2 vs. 15**	-0.4788	-2.092 to 1.134	ns	0.9967
**2 vs. 16**	-0.3628	-2.037 to 1.312	ns	>0.9999
**2 vs. 17**	-0.8892	-2.494 to 0.7151	ns	0.6644
**2 vs. 18**	-0.3508	-2.097 to 1.395	ns	>0.9999
**2 vs. 19**	-1.251	-2.914 to 0.4110	ns	0.2593
**2 vs. 20**	-1.59	-3.177 to -0.002102	*	0.0495

**2 vs. 21**	-1.865	-3.532 to -0.1974	*	0.0215
**3 vs. 4**	0.4925	-0.3693 to 1.354	ns	0.5781
**3 vs. 5**	-0.2132	-2.352 to 1.925	ns	>0.9999
**3 vs. 6**	-0.8474	-2.799 to 1.104	ns	0.9171
**3 vs. 7**	-0.3025	-1.897 to 1.292	ns	>0.9999
**3 vs. 8**	-0.06211	-1.452 to 1.328	ns	>0.9999
**3 vs. 9**	-0.1283	-1.672 to 1.415	ns	>0.9999
**3 vs. 10**	-1.569	-2.832 to -0.3049	**	0.0095
**3 vs. 11**	-0.9497	-2.585 to 0.6851	ns	0.6001
**3 vs. 12**	-1.494	-3.633 to 0.6454	ns	0.3542
**3 vs. 13**	-1.385	-3.457 to 0.6880	ns	0.3853
**3 vs. 14**	-2.081	-3.812 to -0.3493	*	0.0118
**3 vs. 15**	-0.7473	-2.035 to 0.5403	ns	0.6013
**3 vs. 16**	-0.6314	-1.817 to 0.5542	ns	0.7337
**3 vs. 17**	-1.158	-2.466 to 0.1502	ns	0.1086
**3 vs. 18**	-0.6194	-1.712 to 0.4736	ns	0.6345
**3 vs. 19**	-1.52	-3.566 to 0.5262	ns	0.275
**3 vs. 20**	-1.858	-4.077 to 0.3609	ns	0.1525
**3 vs. 21**	-2.133	-3.799 to -0.4677	**	0.0068
**4 vs. 5**	-0.7057	-2.404 to 0.9928	ns	0.9067
**4 vs. 6**	-1.34	-2.217 to -0.4628	**	0.002
**4 vs. 7**	-0.795	-1.598 to 0.008214	ns	0.0534
**4 vs. 8**	-0.5546	-1.276 to 0.1663	ns	0.1995
**4 vs. 9**	-0.6208	-1.808 to 0.5661	ns	0.6934
**4 vs. 10**	-2.061	-4.224 to 0.1012	ns	0.0665
**4 vs. 11**	-1.442	-3.349 to 0.4650	ns	0.2151
**4 vs. 12**	-1.986	-3.682 to -0.2903	*	0.0169
**4 vs. 13**	-1.877	-3.709 to -0.04577	*	0.0433
**4 vs. 14**	-2.573	-4.497 to -0.6496	**	0.0061
**4 vs. 15**	-1.24	-2.504 to 0.02395	ns	0.0561
**4 vs. 16**	-1.124	-2.362 to 0.1143	ns	0.0897
**4 vs. 17**	-1.65	-3.038 to -0.2627	*	0.0161
**4 vs. 18**	-1.112	-2.420 to 0.1959	ns	0.1239
**4 vs. 19**	-2.012	-3.199 to -0.8257	***	0.0008
**4 vs. 20**	-2.351	-3.851 to -0.8507	**	0.0016
**4 vs. 21**	-2.626	-4.340 to -0.9111	**	0.002
**5 vs. 6**	-0.6342	-2.362 to 1.093	ns	0.9737
**5 vs. 7**	-0.08923	-1.456 to 1.278	ns	>0.9999
**5 vs. 8**	0.1511	-0.9206 to 1.223	ns	>0.9999
**5 vs. 9**	0.08494	-1.600 to 1.770	ns	>0.9999
**5 vs. 10**	-1.355	-4.623 to 1.913	ns	0.9212
**5 vs. 11**	-0.7365	-3.181 to 1.708	ns	0.9949
**5 vs. 12**	-1.281	-2.927 to 0.3663	ns	0.2158
**5 vs. 13**	-1.171	-2.928 to 0.5847	ns	0.3697
**5 vs. 14**	-1.867	-4.528 to 0.7933	ns	0.3352
**5 vs. 15**	-0.5341	-2.588 to 1.520	ns	0.999

**5 vs. 16**	-0.4181	-2.679 to 1.843	ns	>0.9999
**5 vs. 17**	-0.9445	-3.131 to 1.242	ns	0.8971
**5 vs. 18**	-0.4061	-2.770 to 1.958	ns	>0.9999
**5 vs. 19**	-1.307	-2.932 to 0.3187	ns	0.1834
**5 vs. 20**	-1.645	-3.292 to 0.002448	ns	0.0505
**5 vs. 21**	-1.92	-3.961 to 0.1213	ns	0.075
**6 vs. 7**	0.5449	-0.6863 to 1.776	ns	0.9056
**6 vs. 8**	0.7853	-0.3764 to 1.947	ns	0.3851
**6 vs. 9**	0.7191	-0.9355 to 2.374	ns	0.9166
**6 vs. 10**	-0.7213	-3.606 to 2.164	ns	0.9996
**6 vs. 11**	-0.1023	-2.161 to 1.956	ns	>0.9999
**6 vs. 12**	-0.6464	-2.784 to 1.491	ns	0.9968
**6 vs. 13**	-0.5373	-2.975 to 1.901	ns	0.9999
**6 vs. 14**	-1.233	-3.270 to 0.8030	ns	0.5586
**6 vs. 15**	0.1	-1.341 to 1.541	ns	>0.9999
**6 vs. 16**	0.216	-1.794 to 2.227	ns	>0.9999
**6 vs. 17**	-0.3103	-2.111 to 1.490	ns	>0.9999
**6 vs. 18**	0.228	-1.901 to 2.357	ns	>0.9999
**6 vs. 19**	-0.6725	-2.668 to 1.323	ns	0.9901
**6 vs. 20**	-1.011	-3.391 to 1.369	ns	0.929
**6 vs. 21**	-1.286	-3.572 to 1.001	ns	0.6634
**7 vs. 8**	0.2403	-0.4323 to 0.9130	ns	0.9792
**7 vs. 9**	0.1742	-1.176 to 1.524	ns	>0.9999
**7 vs. 10**	-1.266	-3.899 to 1.366	ns	0.8273
**7 vs. 11**	-0.6472	-2.511 to 1.217	ns	0.9839
**7 vs. 12**	-1.191	-3.184 to 0.8011	ns	0.5772
**7 vs. 13**	-1.082	-3.292 to 1.128	ns	0.7914
**7 vs. 14**	-1.778	-3.707 to 0.1510	ns	0.0864
**7 vs. 15**	-0.4449	-1.757 to 0.8675	ns	0.9872
**7 vs. 16**	-0.3289	-1.818 to 1.160	ns	>0.9999
**7 vs. 17**	-0.8553	-2.109 to 0.3984	ns	0.3727
**7 vs. 18**	-0.3169	-1.960 to 1.326	ns	>0.9999
**7 vs. 19**	-1.217	-3.113 to 0.6781	ns	0.4728
**7 vs. 20**	-1.556	-3.811 to 0.7000	ns	0.3712
**7 vs. 21**	-1.831	-3.853 to 0.1921	ns	0.0969
**8 vs. 9**	-0.06617	-1.372 to 1.239	ns	>0.9999
**8 vs. 10**	-1.507	-3.985 to 0.9718	ns	0.5157
**8 vs. 11**	-0.8876	-2.716 to 0.9404	ns	0.8002
**8 vs. 12**	-1.432	-3.478 to 0.6144	ns	0.3391
**8 vs. 13**	-1.323	-3.410 to 0.7654	ns	0.4376
**8 vs. 14**	-2.019	-3.560 to -0.4773	**	0.006
**8 vs. 15**	-0.6852	-1.674 to 0.3036	ns	0.3377
**8 vs. 16**	-0.5693	-1.780 to 0.6411	ns	0.8476
**8 vs. 17**	-1.096	-2.238 to 0.04634	ns	0.0654
**8 vs. 18**	-0.5573	-1.902 to 0.7877	ns	0.9217
**8 vs. 19**	-1.458	-3.291 to 0.3751	ns	0.1934

**8 vs. 20**	-1.796	-3.950 to 0.3576	ns	0.1516
**8 vs. 21**	-2.071	-3.820 to -0.3216	*	0.0141
**9 vs. 10**	-1.44	-4.058 to 1.177	ns	0.6737
**9 vs. 11**	-0.8214	-2.865 to 1.222	ns	0.9457
**9 vs. 12**	-1.365	-2.553 to -0.1775	*	0.0172
**9 vs. 13**	-1.256	-3.192 to 0.6793	ns	0.4243
**9 vs. 14**	-1.952	-3.909 to 0.004536	ns	0.0508
**9 vs. 15**	-0.6191	-1.956 to 0.7179	ns	0.8607
**9 vs. 16**	-0.5031	-1.853 to 0.8469	ns	0.9747
**9 vs. 17**	-1.029	-2.064 to 0.005430	ns	0.0518
**9 vs. 18**	-0.4911	-2.032 to 1.050	ns	0.993
**9 vs. 19**	-1.392	-2.365 to -0.4180	**	0.0024
**9 vs. 20**	-1.73	-3.069 to -0.3904	**	0.0063
**9 vs. 21**	-2.005	-4.113 to 0.1034	ns	0.0706
**10 vs. 11**	0.619	-1.573 to 2.811	ns	0.9981
**10 vs. 12**	0.07489	-3.057 to 3.207	ns	>0.9999
**10 vs. 13**	0.184	-2.821 to 3.189	ns	>0.9999
**10 vs. 14**	-0.5121	-2.602 to 1.578	ns	0.9997
**10 vs. 15**	0.8213	-1.486 to 3.128	ns	0.9755
**10 vs. 16**	0.9373	-1.017 to 2.892	ns	0.8301
**10 vs. 17**	0.4109	-1.935 to 2.757	ns	>0.9999
**10 vs. 18**	0.9493	-0.7981 to 2.697	ns	0.6696
**10 vs. 19**	0.04883	-3.030 to 3.128	ns	>0.9999
**10 vs. 20**	-0.2895	-3.565 to 2.987	ns	>0.9999
**10 vs. 21**	-0.5644	-2.528 to 1.400	ns	0.9977
**11 vs. 12**	-0.5441	-2.943 to 1.855	ns	0.9999
**11 vs. 13**	-0.4349	-2.468 to 1.598	ns	0.9999
**11 vs. 14**	-1.131	-3.043 to 0.7813	ns	0.5747
**11 vs. 15**	0.2024	-1.858 to 2.262	ns	>0.9999
**11 vs. 16**	0.3183	-1.350 to 1.987	ns	>0.9999
**11 vs. 17**	-0.208	-1.887 to 1.471	ns	>0.9999
**11 vs. 18**	0.3303	-1.182 to 1.843	ns	0.9999
**11 vs. 19**	-0.5701	-2.991 to 1.851	ns	0.9998
**11 vs. 20**	-0.9084	-3.469 to 1.652	ns	0.9805
**11 vs. 21**	-1.183	-2.870 to 0.5031	ns	0.3354
**12 vs. 13**	0.1091	-1.597 to 1.815	ns	>0.9999
**12 vs. 14**	-0.5869	-3.264 to 2.090	ns	>0.9999
**12 vs. 15**	0.7464	-1.407 to 2.900	ns	0.9842
**12 vs. 16**	0.8624	-1.324 to 3.049	ns	0.9594
**12 vs. 17**	0.336	-1.567 to 2.239	ns	>0.9999
**12 vs. 18**	0.8744	-1.335 to 3.084	ns	0.9517
**12 vs. 19**	-0.02606	-1.201 to 1.149	ns	>0.9999
**12 vs. 20**	-0.3644	-1.453 to 0.7240	ns	0.9907
**12 vs. 21**	-0.6393	-3.051 to 1.772	ns	0.9993
**13 vs. 14**	-0.6961	-3.866 to 2.474	ns	0.9999
**13 vs. 15**	0.6373	-1.936 to 3.211	ns	0.9992

**13 vs. 16**	0.7533	-1.688 to 3.195	ns	0.9935
**13 vs. 17**	0.2269	-2.099 to 2.553	ns	>0.9999
**13 vs. 18**	0.7653	-1.767 to 3.297	ns	0.993
**13 vs. 19**	-0.1352	-2.533 to 2.262	ns	>0.9999
**13 vs. 20**	-0.4735	-2.440 to 1.493	ns	0.9996
**13 vs. 21**	-0.7485	-3.030 to 1.533	ns	0.9882
**14 vs. 15**	1.333	-0.1313 to 2.798	ns	0.0919
**14 vs. 16**	1.449	0.2957 to 2.603	**	0.008
**14 vs. 17**	0.923	-0.7687 to 2.615	ns	0.685
**14 vs. 18**	1.461	0.3304 to 2.592	**	0.0067
**14 vs. 19**	0.5609	-1.399 to 2.520	ns	0.9983
**14 vs. 20**	0.2226	-2.325 to 2.770	ns	>0.9999
**14 vs. 21**	-0.05239	-1.563 to 1.459	ns	>0.9999
**15 vs. 16**	0.116	-1.109 to 1.341	ns	>0.9999
**15 vs. 17**	-0.4104	-1.244 to 0.4236	ns	0.7864
**15 vs. 18**	0.128	-1.090 to 1.346	ns	>0.9999
**15 vs. 19**	-0.7725	-2.601 to 1.056	ns	0.9225
**15 vs. 20**	-1.111	-3.466 to 1.245	ns	0.8452
**15 vs. 21**	-1.386	-3.334 to 0.5628	ns	0.3183
**16 vs. 17**	-0.5264	-1.477 to 0.4246	ns	0.6662
**16 vs. 18**	0.01199	-0.8585 to 0.8825	ns	>0.9999
**16 vs. 19**	-0.8885	-2.651 to 0.8737	ns	0.7961
**16 vs. 20**	-1.227	-3.424 to 0.9700	ns	0.6728
**16 vs. 21**	-1.502	-2.921 to -0.08257	*	0.0331
**17 vs. 18**	0.5384	-0.4855 to 1.562	ns	0.7108
**17 vs. 19**	-0.3621	-2.066 to 1.342	ns	>0.9999
**17 vs. 20**	-0.7004	-2.868 to 1.468	ns	0.9919
**17 vs. 21**	-0.9754	-3.033 to 1.082	ns	0.8407
**18 vs. 19**	-0.9005	-2.753 to 0.9524	ns	0.8174
**18 vs. 20**	-1.239	-3.439 to 0.9619	ns	0.6436
**18 vs. 21**	-1.514	-2.937 to -0.09013	*	0.0323
**19 vs. 20**	-0.3383	-1.449 to 0.7721	ns	0.9965
**19 vs. 21**	-0.6133	-2.808 to 1.581	ns	0.9987
**20 vs. 21**	-0.275	-2.663 to 2.113	ns	>0.9999

*, **,*** denote the significantly different of data when P<0.05, 0.01 and 0.001, respectively.
